# A simple and reliable method for determining kanamycin sensitivity in *Brassica napus*

**DOI:** 10.1093/plphys/kiag244

**Published:** 2026-04-24

**Authors:** Xuelin Wu, Alexandre Miaule, Joanne Chory

**Affiliations:** Howard Hughes Medical Institute, Salk Institute for Biological Studies, 10010 N Torrey Pines Rd, La Jolla, CA 92037, United States; Plant Biology Laboratory, Salk Institute for Biological Studies, 10010 N Torrey Pines Rd, La Jolla, CA 92037, United States; Plant Biology Laboratory, Salk Institute for Biological Studies, 10010 N Torrey Pines Rd, La Jolla, CA 92037, United States; Howard Hughes Medical Institute, Salk Institute for Biological Studies, 10010 N Torrey Pines Rd, La Jolla, CA 92037, United States; Plant Biology Laboratory, Salk Institute for Biological Studies, 10010 N Torrey Pines Rd, La Jolla, CA 92037, United States

Dear Editor,

Floral dipping (Clough and Bent 1998) has enabled the creation of a vast number of transgenic *Arabidopsis* plants and has been essential for understanding gene function. In contrast, the process of transforming *Brassica napus*, a close relative of *Arabidopsis* and an important oil crop, still largely relies on tissue culture–based methods (Ille and Melzer 2025). These protocols are not only labor-intensive and time-consuming, but also prone to a high rate of failure. Although there have been reports of successful transformation of *Brassica* species using floral dipping over the past two decades (Wang et al. 2003; Verma et al. 2008; Li, 2010; Aminedi et al. 2019; Hu et al. 2019), the reported T1 selection methods post-dipping are difficult to adopt, likely due to high sensitivities to growth conditions. Furthermore, the natural color and autofluorescence of the *B. napus* seed coat prevents the use of the more recently developed visible seed coat selection markers (Gao et al. 2016; Liu et al. 2025). The lack of an effective selection also meant that transgene segregation analyses in subsequent generations of *B. napus* require continuous genotyping. This is in stark contrast to the easy selection protocols that are available for *Arabidopsis* studies. Here, we report a highly reliable method for the quick selection of kanamycin-resistant *B. napus* seedlings.

Kanamycin interferes with protein synthesis in the chloroplasts and mitochondria. When *Arabidopsis* seeds are germinated on MS-agar-based medium containing kanamycin, the sensitive seedlings fail to accumulate chlorophyll while the resistant ones turn green. Therefore, it is very easy to determine the kanamycin sensitivity of a given seedling based on the color of its cotyledons. In contrast, kanamycin exerts a much milder effect on *B. napus* (Liu et al. 2013). After one week on kanamycin-containing MS-agar medium, wild-type *B. napus* (c.v. Westar) seedlings developed shorter primary roots than those germinated without selection and lacked lateral roots. Cotyledon expansion was also inhibited at the highest kanamycin concentration tested (Fig. 1A, top panel). The wild-type seedlings germinated in the presence of kanamycin remained green for at least 2 weeks, by which time the seedlings became too large for the Petri dish. In comparison, no clear difference was observed between the transgenic kanamycin-resistant *B. napus* seedlings germinated with and without kanamycin selection on MS-agar (Fig. 1A, bottom panel). However, this subtle phenotypic difference is not sufficiently qualitative to be used for kanamycin selection among a large number of seeds.

**Figure 1:**
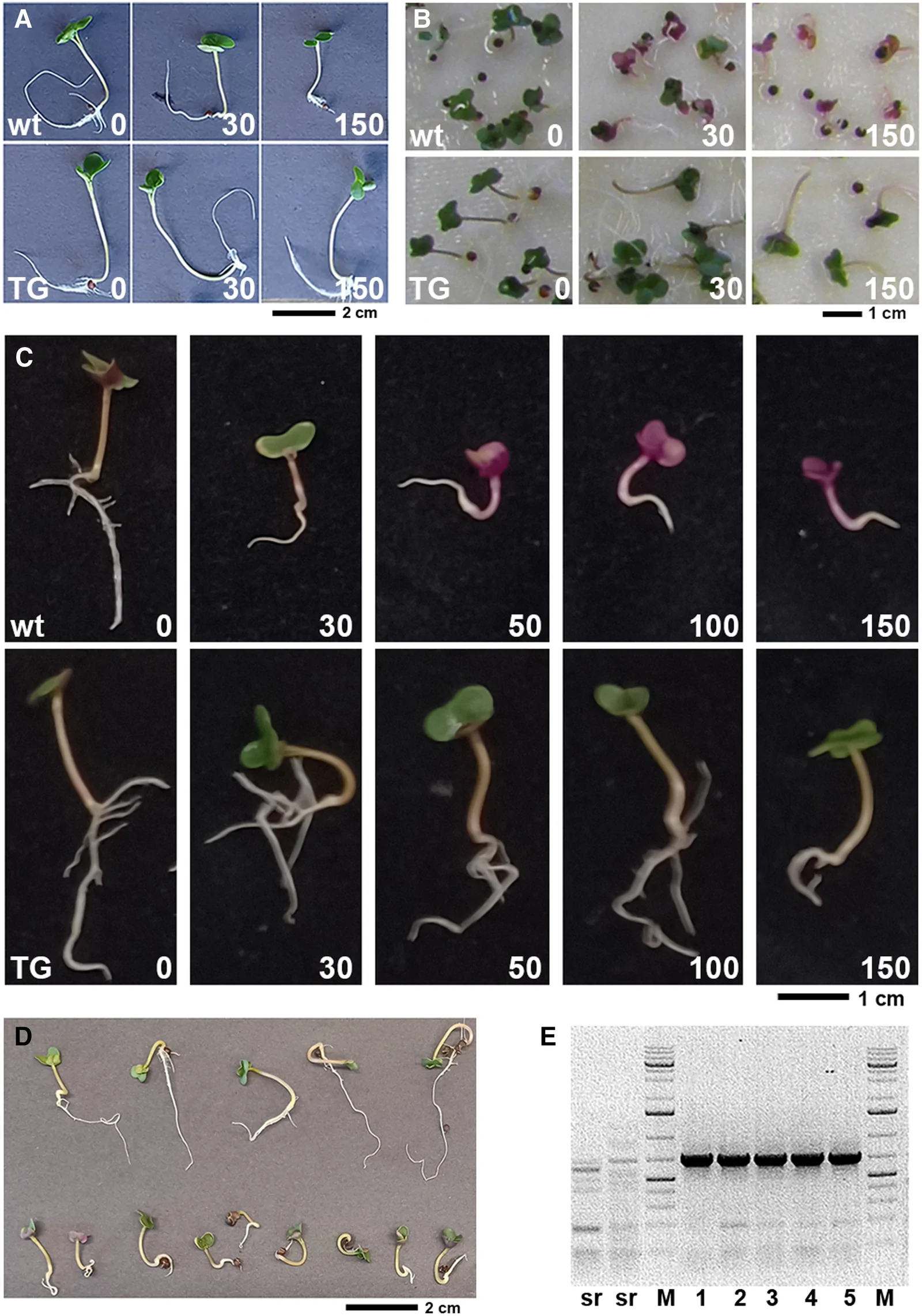
A water-based kanamycin selection method in *Brassica napus*. A) A phenotypic comparison of wild-type (wt, upper panels) and kanamycin-resistant transgenic (TG, lower panels) seedlings grown for 6 days on MS-agar medium containing 0, 30, and 150 μg/mL kanamycin. B) A phenotypic comparison of wild-type (wt, upper panels) and kanamycin-resistant transgenic (TG, lower panels) seedlings grown on water-soaked paper towels containing 0, 30, and 150 μg/mL kanamycin for 6 days. The seeds were placed on top of 6 to 8 layers of paper towel and covered by 1 additional layer. The cover layer was removed after 2 to 3 days once the seeds germinated. C) Phenotype of representative wild-type (wt, top row) and transgenic (TG, bottom row) seedlings after growing for 6 days on paper towels soaked with an increasing concentration of kanamycin. The wild-type root failed to develop in the presence of kanamycin. D) The 5 kanamycin-resistant *B. napus* seedlings were selected from among 60 wild-type seedlings after growing together on paper towel soaked with 30 μg/mL kanamycin for 5 days. Some examples of the wild-type seedlings with short primary roots are shown in the lower row for comparison. Seed density was approximately 70 seeds in a 150 mm petri dish. E) PCR genotyping confirmed the presence of *NPTII* gene in the 5 seedlings with long roots (1 to 5) in (D), and 2 with short roots (sr) were used as the negative control. M, 1 kb plus gene ruler molecular weight marker (Invitrogen). DNA extraction was carried out according to (Klimyuk et al. 1993), and the *NPTII*-specific primers were described in (Verma et al. 2008). All seedlings were grown under 200 μmol m^−2^ s^−1^ of white light, 16 h day at 24 °C/8 h night at 19 °C.

It was previously reported that water-based germination provides an effective condition for hygromycin selection in rice seedlings (Zhang et al. 2012). We therefore tested wild-type *B. napus* seedling response to an increasing concentration of kanamycin on water-soaked paper towels. After 1 week, without kanamycin, wild-type seedlings were fully green. In comparison, anthocyanin accumulation was visible in seedlings germinated with 30 μg/mL kanamycin, and higher kanamycin concentrations prevented chlorophyll accumulation in the cotyledons in most seedlings. A closer examination revealed that while the seedlings grown without selection already developed lateral roots, there was no primary root elongation or lateral root in the ones grown in the presence of kanamycin, regardless of the color of the cotyledons (Fig. 1B and 1C, top panels). These seedlings were unable to adhere to the paper towel, and the green tissue could not be lifted from the surface due to the lack of a physical anchor. Under the same conditions, the kanamycin-resistant seedlings developed green cotyledons at all the concentrations tested. Both primary and lateral roots developed normally when kanamycin concentration did not exceed 100 μg/mL and were able to anchor to the paper towels. Higer kanamycin concentration resulted in significantly shorter primary roots without affecting cotyledon greening (Fig. 1B and 1C, bottom panels). This clear difference in root growth and anchoring provides a qualitative measure for identifying kanamycin-resistant seedlings within a mixed population.

We confirmed the effectiveness of this selection by mixing five kanamycin-resistant seeds into sixty wild-type ones and germinating them on paper towels soaked with kanamycin solution. To reduce any potential toxicity exerted by kanamycin, we chose 30 μg/mL, the lowest effective concentration under our growth conditions. After 5 days, the 2 genotypes were separated by lifting the paper towel and applying a light shake. Only 5 seedlings remained attached to the paper towel because of the presence of elongated primary roots (Fig. 1D, upper row). The presence of the NPTII gene in these 5 seedlings was confirmed using PCR genotyping as described in (Verma et al. 2008) (Fig. 1E). We further tested this selection method on *B. napus* T1 seeds harvested after floral dipping following the procedures described by (Verma et al. 2008) and isolated 7 transgenic lines from approximately one thousand seeds, indicating that it is applicable to T1 selection.

Plant transformation via floral dipping is not only fast and easy but can also largely avoid the chromosomal damage that frequently occurs during callus formation and tissue regeneration. However, the inability to effectively select for transgenic seedlings has been the major obstacle in applying floral dipping to *B. napus*. The method presented here should enable routine selections of transgenic *B. napus* for genetic studies. Although we tested only kanamycin sensitivity of *B. napus* in this study, it is reasonable to expect that this method could also be applied to selections such as hygromycin and glufosinate ammonium (Basta), and to other plant species in which transgenic selection is difficult on standard agar medium. It is worth noting that in applying this method, the effective selection concentration should be first determined based on local growth conditions, especially in light intensity and growth temperature.

## Data Availability

The data underlying in this study is available in the article.

## References

[kiag244-B1] Aminedi R, Dhatwalia D, Jain V, Bhattacharya R. 2019. High efficiency in planta transformation of Indian mustard (Brassica juncea) based on spraying of floral buds. Plant Cell Tissue Organ Cult. 138:229–237. 10.1007/s11240-019-01618-2.

[kiag244-B2] Clough SJ, Bent AF. 1998. Floral dip: a simplified method for Agrobacterium-mediated transformation of Arabidopsis thaliana. Plant J. 16:735–743. 10.1046/j.1365-313x.1998.00343.x. 10069079

[kiag244-B3] Gao X, Chen J, Dai X, Zhang D, Zhao Y. 2016. An effective strategy for reliably isolating heritable and Cas9-free Arabidopsis mutants generated by CRISPR/cas9-mediated genome editing. Plant Physiol. 171:1794–1800. 10.1104/pp.16.00663. 27208253 PMC4936589

[kiag244-B4] Hu D, Bent AF, Hou X, Li Y. 2019. Agrobacterium-mediated vacuum infiltration and floral dip transformation of rapid-cycling Brassica rapa. BMC Plant Biol. 19:246. 10.1186/s12870-019-1843-6. 31182023 PMC6558690

[kiag244-B5] Ille K, Melzer S. 2025. Efficient and versatile rapeseed transformation for new breeding technologies. Plant J. 123:e70330. 10.1111/tpj.70330. 40639408 PMC12245476

[kiag244-B6] Klimyuk VI, Carroll BJ, Thomas CM, Jones JDG. 1993. Alkali treatment for rapid preparation of plant material for reliable PCR analysis. Plant J. 3:493–494. 10.1111/j.1365-313X.1993.tb00169.x. 8220456

[kiag244-B7] Li J, Tan X, Zhu F, Guo J. 2010. A rapid and simple method for Brassica Napus floral-dip transformation and selection of transgenic plantlets. Int J Biol. 2:127–131. 10.5539/ijb.v2n1p127.

[kiag244-B8] Liu J, Yin Y, Li H, Li M. 2013. Effects of antibiotics on seed germination of different rape cultivars. Agric Sci Technol. 14:707–709.

[kiag244-B9] Liu JL et al 2025. Seeing red, selecting true: RUBY-reported seed marker streamlines CRISPR-clean rice breeding. Rice. 18:91. 10.1186/s12284-025-00841-0. 41042306 PMC12495005

[kiag244-B10] Verma SS, Chinnusamy V, Bansa KC. 2008. A simplified floral dip method for transformation of Brassica napus and B. carinata. J Plant Biochem Biotechnol. 17:197–200. 10.1007/BF03263286.

[kiag244-B11] Wang WC, Menon G, Hansen G. 2003. Development of a novel Agrobacterium-mediated transformation method to recover transgenic Brassica napus plants. Plant Cell Rep. 22:274–281. 10.1007/s00299-003-0691-9. 14586552

[kiag244-B12] Zhang C, Li H, Liu B. 2012. A comparison study of hpt and bar as selection marker gene of transgenic rice. Hereditas. 34:1599–1606. 10.3724/sp.j.1005.2012.01599 23262108

